# A single-blind, randomised controlled trial of a physical health nurse intervention to prevent weight gain and metabolic complications in first-episode psychosis: the Physical Health Assistance in Early Psychosis (PHAstER) study

**DOI:** 10.1192/bjo.2022.590

**Published:** 2022-10-18

**Authors:** Brian O'Donoghue, Nathan Mifsud, Emily Castagnini, Alison Langstone, Andrew Thompson, Eoin Killackey, Patrick McGorry

**Affiliations:** Centre for Youth Mental Health, University of Melbourne, Australia; Early Psychosis Prevention and Intervention Centre, Orygen, Australia; and Department of Psychiatry, St Vincent's University Hospital, Ireland; Centre for Youth Mental Health, University of Melbourne, Australia; and Early Psychosis Prevention and Intervention Centre, Orygen, Australia

**Keywords:** Psychotic disorders, antipsychotics, metabolic syndrome, obesity, schizophrenia

## Abstract

**Background:**

Factors that contribute to the early mortality observed in psychotic disorders, specifically obesity, smoking and sedentary behaviour, occur early in the disorder.

**Aims:**

We aimed to determine whether the integration of a physical health nurse in the care of young people with first-episode psychosis could prevent clinically significant weight gain (≥7% body weight). Secondary outcomes included rates of smoking, metabolic syndrome and sedentary behaviour.

**Method:**

In this single-blind, randomised controlled trial, participants who had received under 4 weeks of antipsychotic medication were randomly allocated to either the intervention (addition of a physical health nurse to their care) or treatment as usual (TAU) for 12 weeks.

**Results:**

Of the 77 participants, there were follow-up data for 86.8% (*n* = 33) of the intervention group and 82.1% (*n* = 32) of the TAU group. After 12 weeks, 27.3% of the intervention group experienced clinically significant weight gain compared with 34.4% of the TAU group (odds ratio 0.72, 95% CI 0.25–2.06, *P* = 0.54). After 6 months, 40.7% of the intervention group gained clinically significant weight compared with 44.1% of the TAU group (*P* = 0.79). There was no difference in mean change in weight between groups after 12 weeks (2.6 kg *v*. 2.9 kg, *P* = 0.87) or 6 months (3.6 kg *v*. 4.3 kg, *P* = 0.64). There were no differences in the rates of tobacco smoking cessation, prevalence of metabolic syndrome or physical activity levels.

**Conclusions:**

This intervention failed to prevent the metabolic complications that are highly prevalent in psychotic disorders in the short to medium term, indicating that more intensive interventions are required.

Individuals affected by psychotic disorders have a reduced life expectancy compared with their healthy peers, with individuals with a diagnosis of schizophrenia dying over 15 years earlier.^1^ The main contributor to this early mortality is cardiovascular disease,^2^ arising from higher rates of obesity and diabetes mellitus,^3^ sedentary behavior,^4^ poor diet^5^ and tobacco smoking^6^ in those affected by psychotic disorders. In addition, the commencement of second-generation antipsychotic medication is associated with rapid and significant weight gain, with increases in weight ranging from 4 to 9.2 kg, depending on the specific medication, in the first 3 months.^7^ This translates to between 33 and 61% of people with first-episode psychosis (FEP) experiencing clinically significant weight gain, defined as gaining ≥7% of the original body weight within the first 12 weeks of treatment.^7^

## Prevalence of metabolic complications in FEP

There are nearly 100 systematic reviews and meta-analyses demonstrating a higher prevalence of physical health conditions in people affected by psychotic disorders.^8^ Yet only a handful of interventional studies address this issue in an FEP population,^9^ the clinical population in which prevention of these physical health complications could be possible. An individualised lifestyle and life skills intervention delivered by a nurse, dietician and exercise physiologist managed to reduce the weight gain experienced by young people with FEP over 12 weeks to a mean of 1.8 kg compared with 7.8 kg in another service that offered standard care, translating to only 13% of the intervention group gaining clinically significant weight compared with 75% in standard care.^10^ This important study offered both the framework and hope that cardiometabolic health can be preserved for young people experiencing an FEP. However, the findings have yet to be replicated in a larger, sufficiently powered study. When one component of this intervention, the exercise physiology service, was introduced into a large early intervention for psychosis service, it was found that not all eligible individuals were referred, and for those who were actually referred, attendance and engagement rates were low.^11^ This clinical population may have lowered motivation to engage in physical health interventions, especially those who are experiencing concurrent negative or depressive symptoms.^12,13^

## Interventions aimed at addressing physical health in FEP

A number of physical health interventions were established at the Early Psychosis Prevention and Intervention Centre (EPPIC) within Orygen, a youth mental health service in Melbourne, Australia. These included an exercise physiologist, dietetics, smoking cessation, gym groups and yoga.^11,14,15^ In the early stages of providing treatment to a young person with FEP, the treating team, consisting of a case manager and psychiatrist, have many aspects of care to deliver to the affected individuals and their family or caregiver. To adequately address the physical health of a young person with FEP, the case manager or doctor typically need to make referrals to multiple programmes and provide continual encouragement to the young person to attend these services. Unfortunately, it is a reality of acute clinical services that physical health can be overlooked because of the competing demands of having to attend to acute mental health issues and other needs that may appear more pressing. However, to prevent the onset of these physical health issues, interventions are required from the time of presentation. Therefore, in consultation with young people attending the clinical service, it was deemed a priority to design and evaluate an intervention that facilitated early referral and continued engagement with physical health interventions, with the aim of preventing weight gain and other metabolic complications.

## Objectives

This study evaluated the effectiveness of the addition of a physical health nurse in the care for young people presenting with FEP compared with treatment as usual (TAU), typically consisting of care provided by a case manager and psychiatrist. The Healthy Active Lives declaration sets the target that no more than 25% of young people with FEP will experience clinically significant weight gain, defined as ≥7% body weight.^16^ We hypothesised that the intervention of a dedicated physical health nurse could achieve this target. Therefore, the primary outcome of the study was the prevalence of clinically significant weight gain after 12 weeks. Key secondary hypotheses were whether the effects of the intervention, if any, persisted beyond the end of the intervention period, the mean change of weight between groups, the prevalence of smoking and metabolic syndrome, and levels of physical activity. Finally, we aimed to investigate whether demographic, clinical or physical health factors were associated with the primary outcome.

## Method

### Trial design and randomisation

**Fig. 1:**
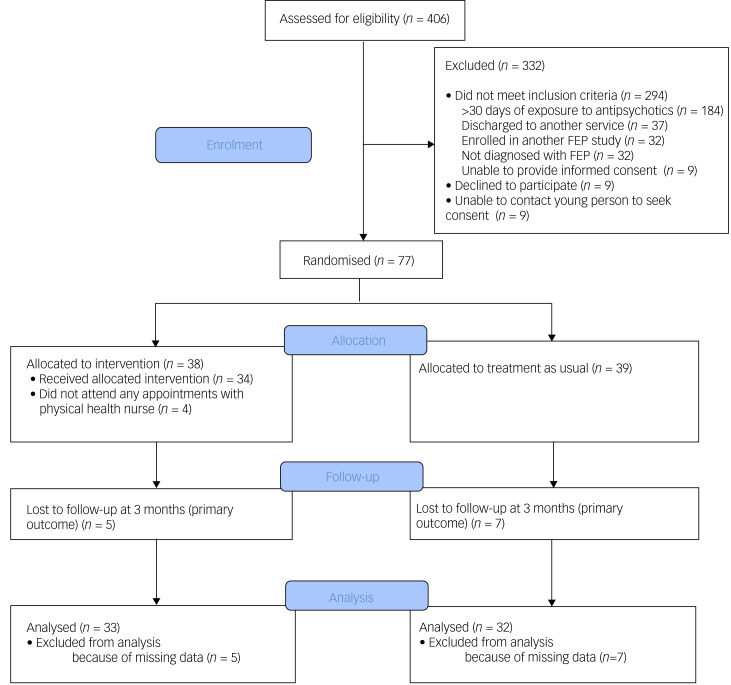
Consolidated Standards of Reporting Trials (CONSORT) diagram of flow of participants. FEP, first-episode psychosis.

This was a single-blind, randomised controlled trial (see Fig. 1 for a study flow diagram). Participants were randomly allocated to have a physical health nurse involved in their care (in addition to the case manager and psychiatrist) or TAU (i.e. only a case manager and psychiatrist). Randomisation was performed by a statistician independent of the study and via an online clinical trials management system. Participants were randomised on a 1:1 ratio and were stratified according to gender and body mass index (classified as either healthy range or overweight/obese).

### Setting

This study was conducted at EPPIC, an early intervention for psychosis service that is part of Orygen, a youth mental health service for young people aged 15–24 years residing in North-West Melbourne, Australia and this covers a total catchment area of over 1 million residents, of whom approximately 200 000 were aged between 15 and 24 years. The EPPIC service consists of seven subteams that each cover a specific catchment area of approximately 30 000 people aged 15–24 years. The seven teams operated out of two main hub sites, and some services had satellite or ‘spoke’ sites. EPPIC provides a psychosocial model of care, and young people are supported to achieve both symptomatic and functional recovery.

### Inclusion and exclusion criteria

Participants were eligible to be included in this study if they were aged 15–24 years and diagnosed with FEP, as defined by DSM-V criteria.^17^ Participants also had to have fewer than 30 days of cumulative exposure of the minimum effective dose of an antipsychotic medication, as defined by the Maudsley Prescribing Guidelines.^18^ Participants also had to have capacity to provide informed consent.

### Methods of recruitment and consent process

Informed consent was obtained by a research assistant following verbal and written information on the study. For participants under 18 years of age, written informed consent was obtained from both the participant and a parent/guardian/caregiver. For any participants aged under 18 years who did not have a parent or guardian, or if they did not wish to have them involved, an assessment as to whether the young person met the mature minor criteria was undertaken by a psychiatrist and, if satisfied, they were permitted to provide consent for themselves. Recruitment commenced in August 2018 and ended in December 2020. Consent was obtained in person; however, from April 2020 onward, it was possible to obtain consent via telehealth facilities because of local COVID-19-related restrictions. Recruitment was paused between 16 March 2020 and 14 April 2020 to revise the protocol and obtain ethical approval for changes that were required to continue the trial during local COVID-19-related restrictions. Participants undertook three assessments and were reimbursed A$50 for each assessment. All participants, regardless of group allocation, received an activity tracker (Fitbit Alta, valued at A$150), which they were permitted to keep after the trial.

### Intervention

Participants allocated to the intervention group had a physical health nurse added to their treating team for 12 weeks, in addition to the case manager and psychiatrist. This intervention was applied at the individual participant level and not at a team level, and so there were participants allocated to different groups who received care from the same EPPIC subteam. The physical health nurse provided an opportunity for weekly contact with the participant, which was initially a combination of both face-to-face contact and telehealth. Following the introduction of local COVID-19-related restrictions, the protocol was changed to having all contact via telehealth.

The intervention was a combination of content for all participants, tailored to the specific needs of each participant. All participants in the intervention group received psychoeducation from the physical health nurse about diet, physical activity and the metabolic side-effects of antipsychotic medication, and were referred to the service exercise physiologist and dietician. The physical health nurse also offered to assist the participant in attending these interventions and could accompany them to the appointments. The exercise physiologist and dietician offered individual and group sessions. The above referrals were made for all participants allocated to the intervention group, and there were additional interventions that participants could be supported in attending if it was deemed that they had specific needs and this related to tobacco smoking and sexual health. When relevant, the physical health nurse provided psychoeducation and supported the appropriate referrals to tobacco smoking cessation services, sexual health services and general practitioners. Participants who smoked tobacco were offered a referral to the state-based smoking cessation support service, Quit Victoria, which provides counselling, motivational interviewing and support for people aiming to reduce or cease smoking. Participants who were sexually active and were not attending an appropriate service were provided with psychoeducation about contraception and sexually transmitted infections, and the participant was supported in accessing the appropriate sexual health services if appropriate. The physical health nurse also conducted the physical health screening and metabolic monitoring. A level of flexibility was incorporated into the role of the physical health nurse, as the overarching aim of the intervention was to facilitate young people with FEP to engage in healthy lifestyle interventions; therefore, if a participant expressed a preference to attend a different gym or sports club, this was facilitated. The level of flexibility was increased when the restrictions to manage the COVID-19 pandemic were implemented, and participants were directed toward exercise that they could undertake at home, such as online work-out videos and smartphone applications. An outline of the physical nurse intervention is provided in the Supplementary material available at https://doi.org/10.1192/bjo.2022.590.

Participants who were allocated to the TAU group (case manager and psychiatrist) still had access to the above interventions and services, but it had to be coordinated or performed by the case manager or psychiatrist directly. The case manager or psychiatrist conducted the physical health screening and metabolic monitoring for participants who were allocated to the TAU group. The physical health nurse did not have any contact with individuals allocated to the TAU group, and did not have contact with participants in the intervention group beyond 12 weeks.

### Primary and secondary outcome

The primary outcome of this study was whether, after the 12-week intervention period, there was a difference in the proportion of young people with FEP who gained ≥7% of their body weight in the intervention group versus the TAU group. A secondary outcome was whether there were any changes in this outcome (≥7% body weight gain) after 6 months. Other secondary outcomes were whether there was a difference between groups in the proportion who experienced clinically significant weight gain after 6 months of follow-up and mean difference in weight, tobacco smoking rates, prevalence of metabolic syndrome and physical activity levels after 12 weeks and 6 months.

### Instruments

Diagnosis was determined with the Structured Clinical Interview for DSM-V Disorders.^17^ Level of physical activity was measured with the Simple Physical Health Questionnaire,^19^ and it was calculated by adding the average daily hours of walking (question 3), doing sport or exercise (question 4) and other physical activities (question 5). The Alcohol, Smoking and Substance Involvement Screening Test was used to determine prevalence of tobacco smoking and other substance use.^20^ To describe the clinical characteristics of the cohort, the Brief Psychiatric Rating Scale was used to measure psychopathology,^21^ together with the Schedule for the Assessment of Negative Symptoms^22^ and the Social Occupational Functioning Assessment Scale (SOFAS).^17^

### Antipsychotic medication dose equivalents and classes of antipsychotic medications

The total dose of antipsychotic medication prescribed before randomisation and for the two periods of baseline to 12-week follow-up and 12-week to 6-month follow-up was determined by converting the doses of antipsychotic medications prescribed to risperidone equivalents, using the Maudsley Prescribing Guidelines.^18^

### Criteria for metabolic syndrome and definition of clinically significant weight gain

The International Diabetes Federation criteria for metabolic syndrome was used to determine the presence of metabolic syndrome^23^ (Table 1), but had to be adapted slightly for the needs of this study. The criterion for central obesity was a waist circumference of ≥94 cm in males and ≥80 cm in females; we did not have information on ethnicity, and therefore different cut-offs according to ethnicity could not be applied. A body mass index of ≥30 kg/m^2^ was also used as satisfying criteria for central obesity. In our study setting, some of the external pathology laboratories did not provide the breakdown of cholesterol to high- or low-density lipoprotein, and therefore a total cholesterol of ≥5.0 mmol/L was also used as one of the criteria for metabolic syndrome. There is not a universal definition of clinically significant weight gain. Although some studies take ≥5% increase in body weight as clinically significant,^24^ we chose ≥7% because this is the criteria specified in the Healthy Active Lives declaration.^16^

**Table 1:** Primary and secondary outcomes at 12-week and 6-month follow-up in the intervention and treatment-as-usual groups

	Baseline		12-week follow-up		6-month follow-up	
	Intervention, *n* = 38	Treatment as usual, *n* = 39	Intervention, *n* = 33	Treatment as usual, *n* = 32	Intervention, *n* = 38	Treatment as usual, *n* = 39
	% (*n*)	% (*n*)	% (*n*)	% (*n*)	% (*n*)	% (*n*)
Primary outcome						
Gained ≥7% body weight			27.3 (9)	34.4 (11)	40.7 (11)	44.1 (15)
Did not gain ≥7% body weight			72.7 (24)	65.6 (21)	59.3 (16)	55.8 (19)
			*χ*^2^ = 0.39 (1), *P* = 0.54		*χ*^2^ = 0.07 (1), *P* = 0.79	
Secondary outcomes						
Weight changes, mean (s.d.)					
Mean weight	71.2 (16.2)	70.7 (15.7)	74.4 (17.0)	74.2 (18.6)	79.0 (17.5)	75.6 (19.8)
	*t* = 0.15, d.f. = 75, *P* = 0.88		*t* = 0.03, d.f. = 63, *P* = 0.97		*t* = 0.71, d.f. = 59, *P* = 0.48	
Mean change in weight			2.7 (4.2)	2.9 (5.5)	3.63 (4.64)	4.32 (6.6)
			*t* = −0.17, d.f. = 63, *P* = 0.87		*t* = −0.46, d.f. = 59, *P* = 0.65	
BMI category, % (*n*)						
Healthy range (18.5–24.9 kg/m^2^)	55.3 (21)	56.4 (22)	48.5 (16)	53.1 (17)	33.3 (9)	52.9 (18)
Overweight (25 –29.9 kg/m^2^)	28.9 (11)	25.6 (10)	30.3 (10)	21.9 (7)	33.3 (9)	23.5 (8)
Obese (≥30 kg/m^2^)	13.2 (5)	15.4 (6)	18.2 (6)	18.8 (6)	29.6 (8)	23.5 (8)
Underweight (≤18.5 kg/m^2^)	2.6 (1)	2.6 (1)	3.0 (1)	6.3 (2)	3.7 (1)	0 (0)
	*χ*^2^ = 0.15 (3), *P* = 0.99		*χ*^2^ = 0.88 (3), *P* = 0.83		*χ*^2^ = 3.3 (3), *P* = 0.35	
Tobacco smoking status, % (*n*)	*n* = 37	*n* = 38	*n* = 31	*n* = 31	*n* = 30	*n* = 27
Daily	40.5 (15)	36.8 (14)	35.5 (11)	32.3 (10)	30.0 (9)	40.7 (11)
Non-smoker or very occasional	59.5 (22)	63.2 (24)	64.5 (20)	67.7 (31)	70.0 (21)	59.3 (16)
	*χ*^2^ = 0.11 (1), *P* = 0.74		*χ*^2^ = 0.07 (1), *P* = 0.78		*χ*^2^ = 0.72 (1), *P* = 0.40	
Change in smoking status, % (*n*)						
Ceased smoking			3.4 (1)	6.7 (2)	20.7 (6)	23.1 (6)
Commenced smoking			3.4 (1)	6.7 (2)	17.2 (5)	30.8 (8)
No change			93.1 (27)	86.7 (26)	62.1 (18)	46.2 (12)
			*χ*^2^ = 0.67(2), *P* = 0.72		*χ*^2^ = 1.73, (2), *P* = 0.42	
Metabolic syndrome, % (*n*)	*n* = 21	*n* = 20	*n* = 17	*n* = 18	*n* = 10	*n* = 12
Present	4.8 (1)	15.0 (3)	17.6 (3)	11.1 (2)	20.0 (2)	25.0 (3)
Not present	95.2 (20)	85.0 (17)	82.4 (14)	88.9 (16)	80.0 (8)	75.0 (9)
	*χ*^2^ = 1.22 (1), *P* = 0.27		*χ*^2^ = 0.31 (1), *P* = 0.58		*χ*^2^ = 0.08 (1), *P* = 0.78	
Physical activity levels, median (IQR)	*n* = 33	*n* = 31	*n* = 26	*n* = 26	*n* = 23	*n* = 28
Daily hours walking/exercising	2.5 (1.0–4.2)	1.3 (0.7–2.0)	2.4 (1.4–4.0)	1.5 (0.4–3.5)	2.20 (1.5–3.8)	2.0 (0.6–5.0)
	*Z* = −2.1, *P* = 0.04		*Z* = −1.51, *P* = 0.13		*Z* = −0.57, *P* = 0.57	
Change in physical activity levels, mean (s.d.)			−0.35 (3.3)	0.41 (5.1)	0.69 (3.2)	1.01 (3.6)
			*t* = −0.59 (43), *P* = 0.56		*t* = −0.31 (42), *P* = 0.76	

Criteria for metabolic syndrome was the presence of central obesity and any two of the following four factors: raised triglycerides (≥1.7 mmol/L), reduced high-density lipoprotein cholesterol (<1.03 mmol/L in males or <1.29 mmol/L in females), hypertension (defined as systolic blood pressure ≥130 mmHg or diastolic blood pressure ≥85 mmHg), and fasting glucose >5.6 mmol/L. BMI, body mass index; IQR, interquartile range.

### Sample size determination

We aimed to recruit 88 participants, as this would provide 80% power to detect a difference of 30% in the proportion of participants who experience clinically significant weight gain at the 95% significance level, allowing for approximately 10% attrition.

### Statistical methods

Data were inspected on scatterplots to determine whether they were parametric or non-parametric, and the appropriate test was then used. *t*-Tests and chi-squared (*χ*^2^) tests were used to determine whether there was a difference between the expected outcomes and the observed outcomes in the two groups for parametric continuous variables and categorical variables, respectively. The non-parametric equivalent (i.e. the Mann–Whitney *U*-test) was used to determine whether the median of two groups differed. Means are presented with the s.d. and medians are presented with the interquartile range (IQR). Binary logistic regression was used to determine whether the predictor variables were associated with the outcome variable, which was the presence of clinically significant weight gain after 12 weeks. We also performed *post hoc* sensitivity analysis to determine whether the effectiveness of the intervention differed during periods without restrictions related to the COVD-19 pandemic and during periods with these restrictions.

### Ethical approval and trial registration

The authors assert that all procedures contributing to this work comply with the ethical standards of the relevant national and institutional committees on human experimentation and with the Helsinki Declaration of 1975, as revised in 2008. This clinical trial was approved by the Melbourne Health Human Research Ethics Committee (approval number HREC/18/MH/77) and prospectively registered with the Australian New Zealand Clinical Trials Registry (identifier ACTRN12618001222235). The protocol for the clinical trial has been published elsewhere.^25^

## Results

### Description of participants and attendance data

A total of 77 participants were recruited. Mean age at time of presentation was 19.4 (±3.4) years. A total of 50.6% (*n* = 39) identified as male, 46.8% (*n* = 36) identified as female and 2.6% (*n* = 2) identified as nonbinary. The majority of young people were single (77.9%, *n* = 60), and 70.1% (*n* = 52) were either in employment or enrolled in education. Nearly half had a diagnosis of a schizophrenia/schizophreniform disorder (45.5%, *n* = 35) and 23.4% (*n* = 18) had a diagnosis of an affective psychotic disorder. The mean SOFAS score was 53.4 (±11.2), which is equivalent to serious impairment in social and occupational functioning. A total of 41.6% (*n* = 32) of the cohort were either overweight or obese at the time of recruitment. There were no between-group differences in demographic and clinical characteristics, as displayed in Table 2. The median number of sessions attended by participants allocated to the intervention with the physical health nurse was 6 (IQR 3–8), and this did not include attendance to other physical health interventions, as the data were not available. There was no difference in the total exposure to antipsychotic medications or the type of antipsychotic medication prescribed either before randomisation or throughout the study period, as displayed in Table 3.

**Table 2:** Baseline demographic, clinical, and physical health characteristics of study participants

	Intervention	Treatment as usual	Test of difference	Total cohort (*N* = 77)
	Mean (s.d.) or % (*n*)	Mean (s.d.) or % (*n*)	*t* (d.f.) or *χ*^2^ (d.f.)	Mean (s.d.) %(*n*)
Demographics				
Age at service entry, years	19.6 (3.9)	19.3 (2.9)	*t* = 0.35 (35)	19.4 (3.4)
Gender, % male	50.01 (19)	51.3 (20)	*χ*^2^ = 0.01 (2)	50.6 (39)
Marital status, % never married	78.9 (30)	76.9 (30)	*χ*^2^ = 0.53 (2)	77.9 (60)
Work status, % in education or employment	73.7 (28)	66.7 (26)	*χ*^2^ = 0.45 (1)	70.1 (54)
Living with family of origin, %	65.8 (25)	69.2 (27)	*χ*^2^ = 0.10 (1)	67.5 (52)
Australian born, %	76.3 (29)	84.6 (33)	*χ*^2^ = 0.84 (1)	80.5 (62)
Illness and clinical features				
DSM-IV diagnosis, %				
Schizophrenia/schizophreniform	39.5 (15)	51.3 (20)	*χ*^2^ = 1.76 (3)	45.5 (35)
affective (bipolar/depressive/ schizoaffective)	28.9 (11)	17.9 (7)		23.4 (18)
Substance-induced psychotic disorder	15.8 (6)	12.8 (5)		14.3 (11)
Delusional/NOS/brief reactive psychosis	15.8 (6)	17.9 (7)		16.9 (13)
Social and occupational functioning	55.2 (11.1)	52.0 (11.3)	*t* = 1.27 (75)	53.4 (11.2)
Tobacco smoking, % daily	40.5 (15)	36.8 (14)	*χ*^2^ = 0.11 (1)	38.7 (29)
At least weekly use of cannabis, %	19.4 (7)	13.2 (5)	*χ*^2^ = 0.54 (1)	16.2 (12)
At least weekly use of amphetamines, %	5.6 (2)	5.6 (2)	*χ*^2^ = 0.01 (1)	5.4 (4)
Psychopathology				
BPRS total score (*n* = 72)	59.6 (12.5)	57.8 (12.8)	*t* = 0.59 (70)	58.7 (12.6)
BPRS positive symptom subscale (*n* = 73)	15.5 (4.3)	15.7 (4.0)	*t* = −0.15 (71)	15.6 (4.1)
SANS (*n* = 60)	14.4 (13.1)	19.0 (14.8)	*t* = −1.26 (58)	16.9 (14.1)
Physical health at presentation				
Weight				70.9 (15.9)
BMI, category, %				
Heathy range (18.5–24.9 kg/m^2^)	55.3 (21)	56.4 (22)	*χ*^2^ = 0.15 (3)	55.8 (43)
Overweight (25.0–29.9 kg/m^2^)	28.9 (11)	25.6 (10)		27.3 (21)
Obese (≥30 kg/m^2^)	13.2 (5)	15.4 (6)		14.3 (11)
Underweight (<18.5 kg/m^2^)	2.6 (1)	2.6 (1)		2.6 (2)
Waist circumference (cm) (*n* = 51)				
Males	89.6 (10.6)	89.6 (12.0)	*t* = 1.24 (20)	81.8 (11.0)
Females	84.5 (10.1)	78.7 (11.6)	*t* = 0.01 (26)	86.2 (11.5)
Hypertensive, % (*n* = 48)	34.8 (8)	44.0 (11)	*χ*^2^ = 0.43 (1)	39.6 (19)

NOS, not otherwise specified; BPRS, Brief Psychiatric Rating Scale; SANS, Schedule for the Assessment of Negative Symptoms; BMI, body mass index.

**Table 3:** Antipsychotic medication exposure and treatment according to group allocation

	Before randomisation			Baseline and 12 weeks			12 weeks and 6 months		
	Intervention	Treatment as usual	Statistical test of difference	Intervention	Treatment as usual	Statistical test of difference	Intervention	Treatment as usual	Statistical test of difference
	Median (IQR)	Median (IQR)	Mann–Whitney *U*	Median (IQR)	Median (IQR)	Mann–Whitney *U*	Median (IQR)	Median (IQR)	Mann–Whitney *U*
Total exposure to antipsychotic medication (risperidone equivalent)	33.3 (21–48.7)	28.0 (4–50)	*Z* = −0.70, *P* = 0.48	176.0 (112–280)	206.0 (157–334)	*Z* = −0.95, *P* = 0.34	243.3 (87.5–392)	196.0 (30–328)	*Z* = −0.68, *P* = 0.50
Categories of antipsychotic medication	% (*n*)	% (*n*)	*χ*^2^ (d.f.), *P-*value	% (*n*)	% (*n*)	*χ*^2^ (d.f.), *P*-value	% (*n*)	% (*n*)	*χ*^2^ (d.f.), *P*-value
SGA only	51.4 (18)	51.4 (19)	0.67 (2), 0.71	51.4 (18)	51.4 (19)	0.41 (2), 0.81	47.1 (16)	51.4 (19)	1.52 (3), 0.68
Partial dopamine agonist only	42.9 (15)	37.8 (14)		37.1 (13)	32.4 (12)		35.3 (12)	27.0 (10)	
SGA and partial dopamine agonist	0 (0)	0 (0)		11.4 (4)	16.2 (6)		8.8 (3)	5.4 (2)	
No antipsychotic medication	5.7 (2)	10.8 (4)		0 (0)	0 (0)		8.8 (3)	16.2 (6)	
Specific medication prescribed,^a^ % (*n*)									
Amisulpride	2.6 (1)	0 (0)		2.6 (1)	0 (0)		5.2 (1)	0 (0)	
Aripiprazole	39.5 (15)	35.9 (14)		44.7 (17)	46.2 (18)		39.4 (15)	33.3 (13)	
Lurasidone	5.3 (2)	5.1 (2)		15.8 (6)	15.4 (6)		13.1 (5)	10.3 (4)	
Olanzapine	7.9 (3)	2.6 (1)		10.5 (4)	7.7 (3)		15.8 (5)	7.7 (3)	
Paliperidone	10.5 (4)	7.7 (3)		13.1 (5)	10.3 (4)		5.3 (2)	10.3 (4)	
Risperidone	21.1 (8)	23.1 (9)		13.2 (5)	30.8 (12)		5.3 (2)	20.5 (8)	
Quetiapine	0 (0)	10.3 (4)		0 (0)	20.5 (8)		5.3 (2)	7.7 (3)	
Clozapine	0 (0)	0 (0)		0 (0)	0 (0)		2.6 (1)	0 (0)	
No antipsychotic medication	5.3 (2)	10.3 (4)		0 (0)	0 (0)		7.9 (3)	15.4 (6)	
Missing data	7.9 (3)	5.1 (2)		7.9 (3)	5.1 (2)		10.5 (4)	5.1 (2)	

IQR, interquartile range; SGA, second-generation antipsychotic.

a. Participants could have been prescribed more than one antipsychotic and hence the total percentage may exceed 100.

### Primary outcome: prevalence of clinically significant weight gain at 12 weeks

Of the 77 participants, follow-up data at the primary outcome endpoint of 12 weeks was available for 84.4% (*n* = 65) of the cohort and there was no difference in the attrition rates between the two groups (13.2% for the intervention compared with 17.9% for TAU; χ^2^ = 0.34, d.f. = 1, *P* = 0.56). A total of 27.3% (*n* = 9) of participants in the physical health nurse intervention group experienced clinically significant weight gain over a period of 12 weeks, compared with 34.4% (*n* = 11) in the TAU group, and this difference was not statistically significant (odds ratio 0.72, 95% CI 0.25–2.06, *P* = 0.54). The primary and secondary outcomes are presented in Table 1.

### Secondary outcomes

#### Prevalence of clinically significant weight gain after 6 months

There were follow-up data for 79.2% (*n* = 61) of the cohort after 6 months (intervention: 71.0%, *n* = 27; TAU: 87.2%, *n* = 34). After 6 months, compared with their weight at baseline, 40.7% (*n* = 11) of the intervention group gained ≥7% of body weight, compared with 44.1% (*n* = 15) of the TAU group (*χ*^2^ = 0.07, d.f. = 1, *P* = 0.79).

#### Changes in weight after 12 weeks and 6 months

After the 12-week intervention period, the mean change in weight in the intervention group was 2.6 kg (±4.2), compared with 2.9 kg (±5.5) in the TAU group, which was not a statistically significant difference (*t* = −0.17, d.f. = 63, *P* = 0.87). At the 6-month follow-up, the mean change in weight in the intervention group was 3.6 kg (±4.6), compared with 4.3 kg (±6.6) in the TAU group, which was not a statistically significant difference (*t* = −0.46, d.f. = 59, *P* = 0.65).

#### Rates of tobacco smoking

After the 12-week intervention, one participant (3.4%) in the intervention group ceased tobacco smoking, compared with two (6.7%) in the TAU group (*χ*^2^ = 0.67, d.f. = 2, *P* = 0.72); however, the same number in each group also commenced smoking. After 6 months, compared with the baseline, 20.7% (*n* = 6) in the intervention group ceased smoking, compared with 23.1% (*n* = 6) in the TAU group (*n* = 6) (*χ*^2^ = 1.73 (2), *P* = 0.42); however, 17.2% (*n* = 5) in the intervention group and 30.8% (*n* = 8) in the TAU group commenced smoking.

#### Prevalence of metabolic syndrome

There was a high level of missing data at follow-up for the prevalence of metabolic syndrome, as displayed in Table 1. From the 12-week follow-up assessment, there were data for 45.5% of participants (intervention *n* = 17, TAU *n* = 18) regarding the prevalence of metabolic syndrome. No difference was found in the prevalence of metabolic syndrome in the intervention and TAU group (17.6 *v*. 11.1%, *χ*^2^ = 0.31 (1), *P* = 0.58). From the 6-month follow-up assessment, there were data available for 28.6% of participants (intervention *n* = 10, TAU *n* = 12), which included individuals excluded from the 12-week analysis because of missing data, There was no difference in the prevalence of metabolic syndrome between groups after 6 months (20 *v*. 25%, *χ*^2^ = 0.08 (1), *P* = 0.78). There were only data available for 12 participants, representing 15.6% of the original cohort, on the prevalence of metabolic syndrome for all three time points (baseline, 12 weeks and 6 months), and hence these results are not presented.

#### Physical activity levels after 12 weeks and 6 months

At baseline, before randomisation, participants allocated to the intervention group were more physically active compared with those in the TAU group, with a median of 2.5 h of physical activity compared with 1.3 h in the TAU group (*Z* = −2.1, *P* = 0.04). At the 12-week and 6-month follow-up, there was no difference in the level of physical activity between groups and in the mean change of physical activity levels at 12 weeks (*t* = −0.59(43), *P* = 0.56) and 6 months (*t* = −0.31(42), *P* = 0.76).

#### Predictors at baseline of clinically significant weight gain after 12 weeks

No demographic, clinical and physical health factors at baseline were associated with the development of clinically significant weight gain after 12 weeks, as displayed in Table 4.

**Table 4:** Demographic, clinical, and physical health predictors of clinically significant weight gain at 3-month follow-up

	*β*	s.e.	Odds ratio	95% CI	*P*-value
Model 1, demographic factors (*n* = 65)					
Age	−0.01	0.09	0.99	0.83–1.18	0.93
Gender	−20.7	40192	−	−	0.99
In education or employment	0.56	0.63	1.75	0.51–6.05	0.38
First-generation migrant	2.01	1.11	7.46	0.84–66.1	0.07
Randomisation	0.37	0.57	1.44	0.47–4.41	0.52
Model 2, clinical factors at baseline (*n* = 51)					
Severity of psychotic symptoms (BPRS psychosis score)	−0.03	0.14	0.81	0.74–1.27	0.83
Total severity of psychopathology (BPRS total score)	0.04	0.05	1.04	0.95–1.14	0.44
Negative symptoms (total SANS score)	−0.05	0.03	0.96	0.90–1.01	0.12
Functioning (SOFAS score)	0.03	0.03	1.03	0.96–1.10	0.44
Diagnosis (non-affective or affective psychosis)	0.45	0.94	1.57	0.25–9.83	0.63
Model 3, physical health factors at baseline (*n* = 52)					
Body mass index	0.08	0.07	1.08	0.94–1.25	0.30
Level of physical activity	0.03	0.09	1.03	0.85–1.23	0.79

BPRS, Brief Psychiatric Rating Scale; SANS, Schedule for the Assessment of Negative Symptoms; SOFAS, Social Occupational Functioning Assessment Scale.

#### *Post hoc* analysis on effectiveness of intervention during COVID-19-related restrictions and without restrictions

There was no difference in the primary outcome for the subgroup of participants who completed the study before the introduction of the COVID-19 pandemic restrictions and the subgroup who were enrolled during the restrictions, and these results are presented in the Supplementary material.

## Discussion

### Summary of key findings

The main finding of this study is that the addition of a physical health nurse for a period of 12 weeks in the care of young people presenting with FEP did not result in the prevention of clinically significant weight gain or other metabolic complications. There were no differences in the secondary outcomes of absolute weight gain, cessation of smoking tobacco or levels of physical activity. Worryingly, the proportion of young people with clinically significant weight gain was over 40% in each group after 6 months.

### Limitations

The findings of this study need to be considered within the limitations. First, this study was underpowered as it did not reach the recruitment target because of a fixed period of recruitment related to the funding of the study. In addition, it is possible that the potential effectiveness of the intervention was overestimated in the sample size estimation, as differences of >20% were anticipated. We also relied on self-reported measures for two of the secondary outcomes – levels of physical activity and tobacco smoking – and carbon dioxide monitors could have provided a more objective measure of this outcome. In addition, during the COVID-19-related restrictions, the proportion of participants who had blood tests decreased, and this meant that we had high levels of missing data to determine the proportion of participants who developed metabolic syndrome. In addition, it is possible and likely that the same case managers and psychiatrists provided care to different young people randomised to both groups, and this could have led to ‘contamination’ for the TAU group, as the case manager and psychiatrists would have been made more aware of the importance of physical health and have been more motivated to address physical health in the young people who were allocated to TAU. However, it is also possible that they became deskilled in making onward referrals to physical health interventions and engaging young people in these interventions, as the physical health nurse took on this role for the participants allocated to the intervention group. Finally, we did not have data on the actual attendance of the physical health interventions, such as exercise physiology, dietetics and smoking cessation (Quit Victoria). The reason for this is that any contact with clinicians, including the above services, should have been recorded on the participants electronic records; however, we found that in practice, this did not regularly occur. Therefore, the absence of any clinical notes or recorded contacts would not equate to non-attendance, and the data were deemed too unreliable to present. There was a level of flexibility to the role of the physical health nurse, and so participants may have been supported in attending physical health interventions outside of the clinical programme, such as a local gym or sports clubs, and it would not have been possible to record these attendances.

### Comparison with previous research and potential explanation for findings

There have only been a small number of studies aimed at preventing antipsychotic-induced weight gain in drug-naïve FEP, and there is evidence that a behavioural intervention,^26^ individualised lifestyle and life skills intervention^10^ and metformin^27^ can attenuate the weight gain typically observed. However, on closer inspection of each of these trials, the weight gain in the TAU group was very high. In the randomised controlled trial of a behavioural intervention, the TAU group gained a mean of 6.8 kg (s.d. ± 4.5) and 78.8% experienced clinically significant weight gain in a 3-month period.^26^ The results were similar in a randomised controlled trial of metformin, in which the TAU group (olanzapine plus placebo) gained 6.9 kg (s.d. ± 4.3), which corresponded to 63.2% experiencing clinically significant weight gain over 12 weeks. In a study evaluating an individualised lifestyle and life skills intervention, the TAU group from the standard service gained a mean of 7.8 kg, and this corresponded to 75% experiencing clinically significant weight gain over 12 weeks.

In our study, the mean weight gain in the TAU was 2.9 kg (±5.5) and 34.4% experienced clinically significant weight gain, which is far below that of the other trials. The findings of the intervention group in our study were also similar to that observed in the aforementioned trials, therefore it needs to be considered that one of the potential reasons for not demonstrating a benefit to the intervention is that the TAU did reasonably well compared with other first-episode cohorts. This study was conducted within a specialist early intervention for psychosis service that already had a strong focus on delivering comprehensive holistic care, including physical health. For example, in addition to the aforementioned physical health interventions, olanzapine is not used as a first-line antipsychotic medication because of its metabolic side-effects, and medications with a lower propensity for weight gain are used instead.^28^ For all of the randomised controlled trials, by consenting to the study, the participants were already interested in addressing physical health. As they were not blinded to group allocation, if they were allocated to the TAU group they may have been more motivated to engage in physical activity, and the activity tracker could have increased this motivation. Considering that this weight gain and other metabolic complications are highly prevalent, challenging to prevent and can have life-long negative consequences, even the modest result of preventing weight gain in 7% may be worth replicating in a sufficiently powered study.

However, although the TAU group may have done better than expected, over 40% of participants in each group gained clinically significant weight within the first 6 months of treatment for an FEP. Worryingly, the rates of metabolic complications are likely to be much higher in services without these interventions.

Another striking finding of the study is that 21.8% of participants ceased smoking by the 6-month follow-up point; however, 23.6% started smoking tobacco on a daily basis. This highlights that in addition to the need to support young people with FEP to stop smoking, there is also a need to encourage them not to commence it in the period after diagnosis with FEP. The high prevalence of tobacco smoking in the years before presentation with FEP has been well established,^6^ but less is known about the reasons why young people start smoking in the first 6 months after presentation. These findings indicate that a preventative approach must also be taken in relation to tobacco smoking in this clinical cohort, as well as providing interventions to support people to cease smoking.^29^

### Further research

The findings of this study are disappointing, as interventions that can prevent the onset of physical health complications in early psychosis are urgently needed. In hindsight, there are some aspects of the study design that may have hindered determining the full effectiveness of the intervention.

First, the intervention was limited according to time (12 weeks), as opposed to the number of sessions. We had identified the first 12 weeks as the highest risk period for the development of weight gain and other metabolic complications, and so decided that this should represent the intervention period. However, another way of delivering the intervention would have been to limit it to 12 sessions. The median number of sessions attended was six and, anecdotally, we found that participants had other commitments that meant that some sessions had to be cancelled, and the limited time period meant that it was difficult to reschedule. Additionally, if this intervention was to be implemented into routine clinical practice, it would likely be limited to the number of sessions as opposed to time, similar to the chronic disease management plan in Australia, in which people can access a determined number of sessions with allied health professions per year.

Second, lifestyle interventions focus on changing behaviour and attitudes, and it can take time for an individual to integrate these changes into aspects of their daily routine. As a result, the effects of these interventions may be more insidious, and therefore not captured within the follow-up assessments conducted within the study. However, if the impact of these interventions was gradual and observed in the medium or long term, this still does not negate the need to develop interventions that prevent the rapid and significant weight gain that occurs in the early phases of a psychotic disorder.

These intensive interventions could consist of more intensive physical health interventions, such as high-intensity interval training (HIIT), which has been demonstrated to be effective in reducing the waist circumference of people with psychotic disorders who are already overweight.^30^ The potential effects of HIIT in preventing the physical health complications in an FEP cohort has not yet been explored. In this study, we chose the intervention to be a physical health nurse because the nurse could conduct the screening and has the skills to coordinate care across a number of disciplines and specialities, e.g. dietetics, sexual health and discussions about medications. The preliminary work on evaluating the effectiveness of peer workers leading physical health interventions and encouraging positive lifestyle changes has been inconclusive,^31^ but it does represent another option that could be evaluated further.

### Clinical implications

Therefore, the findings of this trial should still encourage early intervention services to adopt physical health interventions. Considering that this clinical population can be sedentary for at least 11 h of their waking day,^4,32^ very intensive interventions are warranted. There is good evidence supporting the use of metformin to reduce weight gain associated with antipsychotic medication,^33^ and as such, it is recommended in those who have experienced clinically significant weight gain.^34^ However, the evidence is less clear about the use of metformin in preventing these metabolic side effects and being used at the time of commencement of antipsychotic medication.^34^ Considering the high levels of weight gain observed in all participants of this study, even those who received an intensive lifestyle intervention, more research is required to evaluate the effectiveness of using medications such as metformin in preventing weight gain and other metabolic complications.

In conclusion, the physical health nurse intervention in addition to a standard early intervention treatment package did not prevent the weight gain or other metabolic complications that are highly prevalent in people affected by psychotic disorders. These findings highlight that these physical health complications are dramatic and occur rapidly. There is an urgent need to develop and evaluate preventative interventions.

## Data Availability

The data that support the findings of this study are available on request from the corresponding author (B.O.). Ethical approval was not obtained to make the data publicly available.

## Supplementary material

For supplementary material accompanying this paper visit http://doi.org/10.1192/bjo.2022.590. click here to view supplementary material

## References

[1] Hjorthøj C, Stürup AE, McGrath JJ, Nordentoft M. Years of potential life lost and life expectancy in schizophrenia: a systematic review and meta-analysis. Lancet Psychiatry 2017; 4(4): 295–301. 28237639 10.1016/S2215-0366(17)30078-0

[2] Ringen PA, Engh JA, Birkenaes AB, Dieset I, Andreassen OA. Increased mortality in schizophrenia due to cardiovascular disease - a non-systematic review of epidemiology, possible causes, and interventions. Front Psychiatry 2014; 5: 137. 25309466 10.3389/fpsyt.2014.00137 PMC4175996

[3] Annamalai A, Kosir U, Tek C. Prevalence of obesity and diabetes in patients with schizophrenia. World J Diabetes 2017; 8(8): 390–6. 28861176 10.4239/wjd.v8.i8.390 PMC5561038

[4] Stubbs B, Williams J, Gaughran F, Craig T. How sedentary are people with psychosis? A systematic review and meta-analysis. Schizophr Res 2016; 171(1–3): 103–9. 26805414 10.1016/j.schres.2016.01.034

[5] Aucoin M, LaChance L, Cooley K, Kidd S. Diet and psychosis: a scoping review. Neuropsychobiology 2020; 79(1): 20–42. 30359969 10.1159/000493399

[6] Myles N, Newall HD, Curtis J, Nielssen O, Shiers D, Large M. Tobacco use before, at, and after first-episode psychosis: a systematic meta-analysis. J Clin Psychiatry 2012; 73(4): 468–75. 22579146 10.4088/JCP.11r07222

[7] Alvarez-Jimenez M, Gonzalez-Blanch C, Crespo-Facorro B, Hetrick S, Rodriguez-Sanchez JM, Perez-Iglesias R, Antipsychotic-induced weight gain in chronic and first-episode psychotic disorders: a systematic critical reappraisal. CNS Drugs 2008; 22(7): 547–62. 18547125 10.2165/00023210-200822070-00002

[8] Firth J, Siddiqi N, Koyanagi A, Siskind D, Rosenbaum S, Galletly C, The lancet psychiatry commission: a blueprint for protecting physical health in people with mental illness. Lancet Psychiatry 2019; 6(8): 675–712. 31324560 10.1016/S2215-0366(19)30132-4

[9] Fouhy F, Cullen W, O'Connor K. Physical health interventions for patients who have experienced a first episode of psychosis: a narrative review. Ir J Psychol Med 2021; 38(1): 62–75. 32985401 10.1017/ipm.2020.92

[10] Curtis J, Watkins A, Rosenbaum S, Teasdale S, Kalucy M, Samaras K, Evaluating an individualized lifestyle and life skills intervention to prevent antipsychotic-induced weight gain in first-episode psychosis. Early Interv Psychiatry 2016; 10(3): 267–76. 25721464 10.1111/eip.12230

[11] Pearce M, Foote L, Brown E, O'Donoghue B. Evaluation of an exercise physiology service in a youth mental health service. Ir J Psychol Med 2021; 38(1): 56–61. 10.1017/ipm.2020.91 32811583

[12] Lyne J, O'Donoghue B, Roche E, Renwick L, Cannon M, Clarke M. Negative symptoms of psychosis: a life course approach and implications for prevention and treatment. Early Interv Psychiatry 2018; 12(4): 561–71. 29076240 10.1111/eip.12501

[13] Herniman SE, Allott K, Phillips LJ, Wood SJ, Uren J, Mallawaarachchi SR, Depressive psychopathology in first-episode schizophrenia spectrum disorders: a systematic review, meta-analysis and meta-regression. Psychol Med 2019; 49(15): 2463–74. 31524121 10.1017/S0033291719002344

[14] McGuire D, Shannon A, Somaiya J, Brown E, O'Donoghue B. A pilot study of a yoga intervention for the treatment of anxiety in young people with early psychosis. Early Interv Psychiatry 2022; 16(2): 200–4. 10.1111/eip.13151 33929083

[15] Crlenjak C, Nicholl H. Physical and Mental Health. Guidance, Resource and Tools for Prevention and Intervention in Cardiometabolic and Sexual Health Issues. Orygen, the National Centre of Excellence in Youth Mental Health, 2017 (https://www.orygen.org.au/Training/Resources/Physical-and-sexual-health/Clinical-practice-points/Physical-mental-health).

[16] International Physical Health in Youth (iphYs) Working Group. *Healthy Active Lives (HeAL) Consensus Statement*. iphYs, 2013 (https://www.iphys.org.au/).

[17] American Psychiatric Association. Diagnostic and Statistical Manual of Mental Disorders (5th edn). American Psychiatric Association, 2013.

[18] Taylor D, Barnes TR, Young AH. The Maudsley Prescribing Guidelines in Psychiatry (13th edn). Wiley Blackwell, 2018.

[19] Rosenbaum S, Morell R, Abdel-Baki A, Ahmadpanah M, Anilkumar TV, Baie L, Assessing physical activity in people with mental illness: 23-country reliability and validity of the Simple Physical Activity Questionnaire (SIMPAQ). BMC Psychiatry 2020; 20: 108. 32143714 10.1186/s12888-020-2473-0 PMC7060599

[20] Newcombe DA, Humeniuk RE, Ali R. Validation of the World Health Organization Alcohol, Smoking and Substance Involvement Screening Test (ASSIST): report of results from the Australian site. Drug Alcohol Rev 2005; 24(3): 217–26. 16096125 10.1080/09595230500170266

[21] Overall JE, Gorham DR. The Brief Psychiatric Rating Scale. Psychol Rep 1962; 10(3): 799–812.

[22] Andreasen NC. The Scale for the Assessment of Negative Symptoms. University of Iowa, 1983.

[23] International Diabetes Federation (IDF). *The IDF Consensus Worldwide Definition of the Metabolic Syndrome*. IDF, 2006 (https://www.idf.org/e-library/consensus-statements/60-idfconsensus-worldwide-definitionof-the-metabolic-syndrome.html).

[24] Domecq JP, Prutsky G, Leppin A, Sonbol MB, Altayar O, Undavalli C, Drugs commonly associated with weight change: a systematic review and meta-analysis. J Clin Endocrinol Metab 2015; 100(2): 363–70. 25590213 10.1210/jc.2014-3421 PMC5393509

[25] O'Donoghue B, Mifsud NG, Tindall RM, Foote L, Hartmann JA, Obst K, Physical health assistance in early recovery of psychosis: study protocol for a randomized controlled trial. Early Interv Psychiatry 2020; 14(5): 587–93. 31643142 10.1111/eip.12884

[26] Alvarez-Jiménez M, González-Blanch C, Vázquez-Barquero JL, Pérez-Iglesias R, Martínez-García O, Pérez-Pardal T, Attenuation of antipsychotic-induced weight gain with early behavioral intervention in drug-naive first-episode psychosis patients: a randomized controlled trial. J Clin Psychiatry 2006; 67(8): 1253–60. 16965204 10.4088/jcp.v67n0812

[27] Wu RR, Zhao JP, Guo XF, He YQ, Fang MS, Guo WB, Metformin addition attenuates olanzapine-induced weight gain in drug-naive first-episode schizophrenia patients: a double-blind, placebo-controlled study. Am J Psychiatry 2008; 165(3): 352–8. 18245179 10.1176/appi.ajp.2007.07010079

[28] Nguyen T, Seiler N, Maguire J, Sizer H, McGorry P, Brown E, Reduction in the prescription of olanzapine as a first-line treatment for first episode psychosis following the implementation of clinical practice guidelines. Schizophr Res 2020; 215: 469–70. 31493968 10.1016/j.schres.2019.08.027

[29] Siskind DJ, Wu BT, Wong TT, Firth J, Kisely S. Pharmacological interventions for smoking cessation among people with schizophrenia spectrum disorders: a systematic review, meta-analysis, and network meta-analysis. Lancet Psychiatry 2020; 7(9): 762–74. 32828166 10.1016/S2215-0366(20)30261-3

[30] Romain AJ, Fankam C, Karelis AD, Letendre E, Mikolajczak G, Stip E, Effects of high intensity interval training among overweight individuals with psychotic disorders: a randomized controlled trial. Schizophr Res 2019; 210: 278–86. 30595443 10.1016/j.schres.2018.12.021

[31] Stubbs B, Williams J, Shannon J, Gaughran F, Craig T. Peer support interventions seeking to improve physical health and lifestyle behaviours among people with serious mental illness: a systematic review. Int J Ment Health Nurs 2016; 25(6): 484–95. 27600483 10.1111/inm.12256

[32] O'Donoghue B, Castagnini E, Langstone A, Mifsud N, Thompson A, Killackey E, Sedentary behaviour in young people presenting with a first episode of psychosis before and during the covid-19 pandemic restrictions. Schizophr Res 2021; 233: 31–3. 34225024 10.1016/j.schres.2021.06.006

[33] Fitzgerald I, O'Connell J, Keating D, Hynes C, McWilliams S, Crowley EK. Metformin in the management of antipsychotic-induced weight gain in adults with psychosis: development of the first evidence-based guideline using GRADE methodology. Evid Based Ment Health 2022; 25(1): 15–22. 10.1136/ebmental-2021-300291 PMC8788031 34588212

[34] Correll CU, Sikich L, Reeves G, Riddle M. Metformin for antipsychotic-related weight gain and metabolic abnormalities: when, for whom, and for how long? Am J Psychiatry 2013; 170(9): 947–52. 24030606 10.1176/appi.ajp.2013.13060771

