# Supplementary material for: A single-blind, randomised controlled trial of a physical health nurse intervention to prevent weight gain and metabolic complications in first-episode psychosis: the Physical Health Assistance in Early Psychosis (PHAstER) study
Source: BJPsych Open. 2022 Oct 18;8(6):e189. doi: 10.1192/bjo.2022.590 (PMC9634606; doi:10.1192/bjo.2022.590)
Supplement: Supplementary file 1 [file bjosup.zip › S2056472422005907sup001.docx]

*Post-hoc analysis on effectiveness of intervention during COVID-19 restrictions and without restrictions*

The analysis pertaining to the primary outcome was repeated separately for participants recruited during the periods of 01.08.18 to 06.01.20 and 09.11.20 to 31.12.20, as they would not have been subject to any COVID-19 restrictions during their intervention period and also the period of 30.03.20 to 08.11.20 in which restrictions were in place.

The primary outcome results were similar for participants whose 12 week intervention period was unaffected by the COVID-19 pandemic restrictions with 26.3% (n=5) of the intervention group gaining clinically significant weight gain compared to 31.3% (n=5) in the TAU group (χ^2^=0.10, df=1,p=.75). Of those, who were recruited during the period when restrictions were in place, 30.8% (n=4) of the intervention group gained clinically significant weight gain compared to 40.0 (n=6) in the TAU group (χ^2^=0.26,df=1,p=.61). In regards to the primary outcome in the total group, 28.6% (n=10) gained clinically significant weight in the total cohort who did not have COVID-19 pandemic restrictions during their 12 week intervention period compared to 35.7% (n=10) of the total cohort who were subject to pandemic restrictions (χ^2^=0.37, df=1,p=.55).
