# Supplementary material for: A single-blind, randomised controlled trial of a physical health nurse intervention to prevent weight gain and metabolic complications in first-episode psychosis: the Physical Health Assistance in Early Psychosis (PHAstER) study
Source: BJPsych Open. 2022 Oct 18;8(6):e189. doi: 10.1192/bjo.2022.590 (PMC9634606; doi:10.1192/bjo.2022.590)
Supplement: Supplementary file 1 [file bjosup.zip › S2056472422005907sup002.docx]

**Appendix 1: Outline of the Physical health nurse intervention.**

**Session 1**

The first session will involve an Introduction to the PHAstER intervention, how to contact the physical health nurse, limitations to confidentiality, team approach to care.  Information on the persons physical health and the health services are currently involved in addition to Orygen, i.e. GP/ allied health/ dental/ optician/ specialists / pharmacy.

In the first session a full history in regards to physical health will taken and will include: (using the HEADSS format) Social history, current diagnoses (MH), family history, past medical history, current  medical history, medication and side effects, allergies/ alerts, immunisation history (can be checked on PRODA), dental, eye care, diet, alcohol and drug use, smoking cessation, sexual/ reproductive health, exercise, lifestyle and sleep hygiene. Metabolic monitoring will be conducted.

**Session 2**

This session will focus on psychoeducation on the benefits of exercise (mental health and physical health well being), recommended exercise guidelines (better health or Department of Health), the screening instrument for undertaking exercise will be used. Opportunities to exercise within the home will be explored, in addition to exercise outdoors while adhering to social distancing principles (when the covid-19 pandemic restrictions were in place). Also, motivation and barriers to undertaking exercise will be explored. Referrals to the exercise physiology service and dietetics service will be made at this point.

**Session 3**

This session will focus on how exercise can be undertaken in a confined space and how technology could be used to add this exercise, e.g. the body coach, couch to 5k, High Intensity Interval training, Yoga/ pilates on Youtube or apps. It will also be explored how some video games, that involve physical movement, such as the Nintendo Wii or Sony Playstation could be option.  Also other methods of motivation, such as pairing up with a friend (via video link) could develop a social component to the exercise.

**Session 4**

This session will focus on diet and the Orygen resource will be shared with the young person and the different food groups will be discussed and strategies to have a more nutritious, healthy diet will be discussed. Current diet will be reviewed. Also methods to obtain more specialized dietetics care will be discussed – such as via the MBS Chronic Disease management plan. Metabolic monitoring will be conducted.

**Session 5**

This session will focus on psychoeducation and assessment of side-effects of antipsychotic medication, which can include increased appetite, slowing of the metabolism, sedation, agitation, which can all effect the ability to engage in exercise. The participant will be supported in discussing these issues with their treating doctor.  

**Session 6**

This session will serve as an opportunity to review current levels of exercise and problem solve any potential barriers to engaging in physical health activities.

**Session 7**

This session will focus on sexual and reprodcutive health. It will be determined whether the participant has received Immunisations for prevention ( hep B, Gardasil), a sexual health history will be taken and a discussion around screening for STIS/ BBVs / HPV will occur. The participant will be informed about how to access things contraception and how to access the Family planning Victoria helpline.

**Session 8**

This session will review whether the young person regularly attends their GP & allied health and if they do not have a regular GP, they will be assisted in finding one and arranging an appointment.  Metabolic monitoring will be conducted.

**Sessions 9 – 11**

These sessions will be tailored specifically to the individual in regards to what is the most pertinent aspect of their physical health that needs to be addressed. The level of exercise will also be assessed and problem solving to overcome any barriers if present.

**Session 12**

This will be a closing session and the participant will be assisted in making plans as to how they can continue to manage their own physical health without the assistance of the physical health nurse. A list of resources will be provided to the participant. Metabolic monitoring will be conducted.
